# Molecular surveillance of Kelch 13 polymorphisms in *Plasmodium falciparum* isolates from Kenya and Ethiopia

**DOI:** 10.1186/s12936-023-04812-y

**Published:** 2024-01-29

**Authors:** Brook Jeang, Daibin Zhong, Ming-Chieh Lee, Harrysone Atieli, Delenasaw Yewhalaw, Guiyun Yan

**Affiliations:** 1 Program in Public Health, University of California Irvine, Irvine, CA USA; 2 School of Public Health and Community Development, Maseno University, Kisumu, Kenya; 3 International Center of Excellence for Malaria Research, Tom Mboya University College, Homa Bay, Kenya; 4 School of Medical Laboratory Sciences, Faculty of Health Sciences, Jimma University, Jimma, Ethiopia; 5 Tropical and Infectious Diseases Research Center, Jimma University, Jimma, Ethiopia

**Keywords:** Malaria, *Plasmodium falciparum*, Artemisinin resistance, Africa

## Abstract

**Background:**

Timely molecular surveillance of *Plasmodium falciparum kelch 13* (*k13*) gene mutations is essential for monitoring the emergence and stemming the spread of artemisinin resistance. Widespread artemisinin resistance, as observed in Southeast Asia, would reverse significant gains that have been made against the malaria burden in Africa. The purpose of this study was to assess the prevalence of *k13* polymorphisms in western Kenya and Ethiopia at sites representing varying transmission intensities between 2018 and 2022.

**Methods:**

Dried blood spot samples collected through ongoing passive surveillance and malaria epidemiological studies, respectively, were investigated. The *k13* gene was genotyped in *P. falciparum* isolates with high parasitaemia: 775 isolates from four sites in western Kenya (Homa Bay, Kakamega, Kisii, and Kombewa) and 319 isolates from five sites across Ethiopia (Arjo, Awash, Gambella, Dire Dawa, and Semera). DNA sequence variation and neutrality were analysed within each study site where mutant alleles were detected.

**Results:**

Sixteen Kelch13 haplotypes were detected in this study. Prevalence of nonsynonymous *k13* mutations was low in both western Kenya (25/783, 3.19%) and Ethiopia (5/319, 1.57%) across the study period. Two WHO-validated mutations were detected: A675V in three isolates from Kenya and R622I in four isolates from Ethiopia. Seventeen samples from Kenya carried synonymous mutations (2.17%). No synonymous mutations were detected in Ethiopia. Genetic variation analyses and tests of neutrality further suggest an excess of low frequency polymorphisms in each study site. Fu and Li’s F test statistic in Semera was 0.48 (P > 0.05), suggesting potential population selection of R622I, which appeared at a relatively high frequency (3/22, 13.04%).

**Conclusions:**

This study presents an updated report on the low frequency of *k13* mutations in western Kenya and Ethiopia. The WHO-validated R622I mutation, which has previously only been reported along the north-west border of Ethiopia, appeared in four isolates collected from eastern Ethiopia. The rapid expansion of R622I across Ethiopia signals the need for enhanced monitoring of the spread of drug-resistant *P. falciparum* parasites in East Africa. Although ACT remains currently efficacious in the study areas, continued surveillance is necessary to detect early indicators of artemisinin partial resistance.

**Supplementary Information:**

The online version contains supplementary material available at 10.1186/s12936-023-04812-y.

## Background

Efficacious antimalarial drugs are mainstays of malaria control and elimination programs. Widespread resistance to chloroquine (CQ) and sulfadoxine-pyrimethamine (SP) led to the adoption of artemisinin-based combination therapy (ACT) as the first- and second-line treatments for uncomplicated *Plasmodium falciparum* malaria and chloroquine-resistant *Plasmodium vivax* malaria in most malaria-endemic regions at the turn of the twenty-first century. Within less than a decade, resistance to artemisinins and partner drugs emerged in Southeast Asia. Historically, resistance to CQ and SP arose in Southeast Asia and spread to the African continent, leading to increases in malaria-related deaths. Africa disproportionately shoulders the global burden of malaria [1]. The spread of ACT resistance to Africa would result in devastating human health and economic costs, as there is currently no new class of antimalarials that is suitable for immediate, large-scale implementation as an alternative to ACT [2].

Genetic markers that are associated with drug resistance serve as valuable tools for assessing the geographic origins of resistance and tracking the spread of resistance. The identification of nonsynonymous single nucleotide polymorphisms (SNPs) in a gene encoding the Kelch propeller domain on *P. falciparum* chromosome 13 (*PfKelch13*, or *Pfk13*) as molecular markers of artemisinin partial resistance has greatly facilitated global surveillance of artemisinin resistance [3]. Over 200 nonsynonymous SNPs in the *k13* gene have been reported worldwide. However, *k13* mutations have differential effects on clearance phenotypes, and not all nonsynonymous SNPs indicate artemisinin partial resistance. Extensive clinical and laboratory evidence are required for SNPs to be validated as molecular markers of artemisinin partial resistance [4]. Each newly discovered *k13* mutation must exhibit both significant association with delayed parasite clearance and > 1% survival in ring-stage survival assays to be classified as a validated *k13* marker by the World Health Organization (i.e., WHO-validated). A mutation that meets only one of these two criteria is considered a candidate resistance marker, while a mutation that meets neither of these criteria is considered neutral [4, 5].

Nonsynonymous *k13* mutations are currently relatively rare in Africa, and ACT remains efficacious across the continent [6]. However, partial artemisinin resistance and signs of resistance to ACT partner drugs have been identified, mainly in East Africa [7]. Validated *k13* mutations have been detected in Uganda, Rwanda, and Tanzania, raising alarm in neighbouring East African countries [8–10]. In both Kenya and Ethiopia, CQ was replaced by SP for the treatment of uncomplicated *P. falciparum* malaria in 1998, followed by yet another policy change from SP to ACT in 2004. The switch to ACT was widely implemented in Kenya in 2006 when artemether-lumefantrine (AL) was made available in government hospitals, while rollout of AL across Ethiopia began in 2005 [11, 12]. Studies that have been conducted to investigate changes in *k13* mutant allele frequency and diversity in Kenya [13–17] and Ethiopia [18–21] have largely focused on samples collected within the first ten years after the introduction of ACT.

The WHO recommends testing the efficacy of first-line antimalarial treatments at least once every 24 months at sentinel sites to monitor the emergence, importation, and spread of resistant alleles and to ensure effective case management. To this end, dried blood spot samples collected through ongoing passive surveillance and malaria epidemiological studies were leveraged to assess the prevalence of *k13* polymorphisms in western Kenya and Ethiopia at sites representing varying transmission intensities between 2018 and 2022.

## Methods

### Study sites

**Fig. 1:**
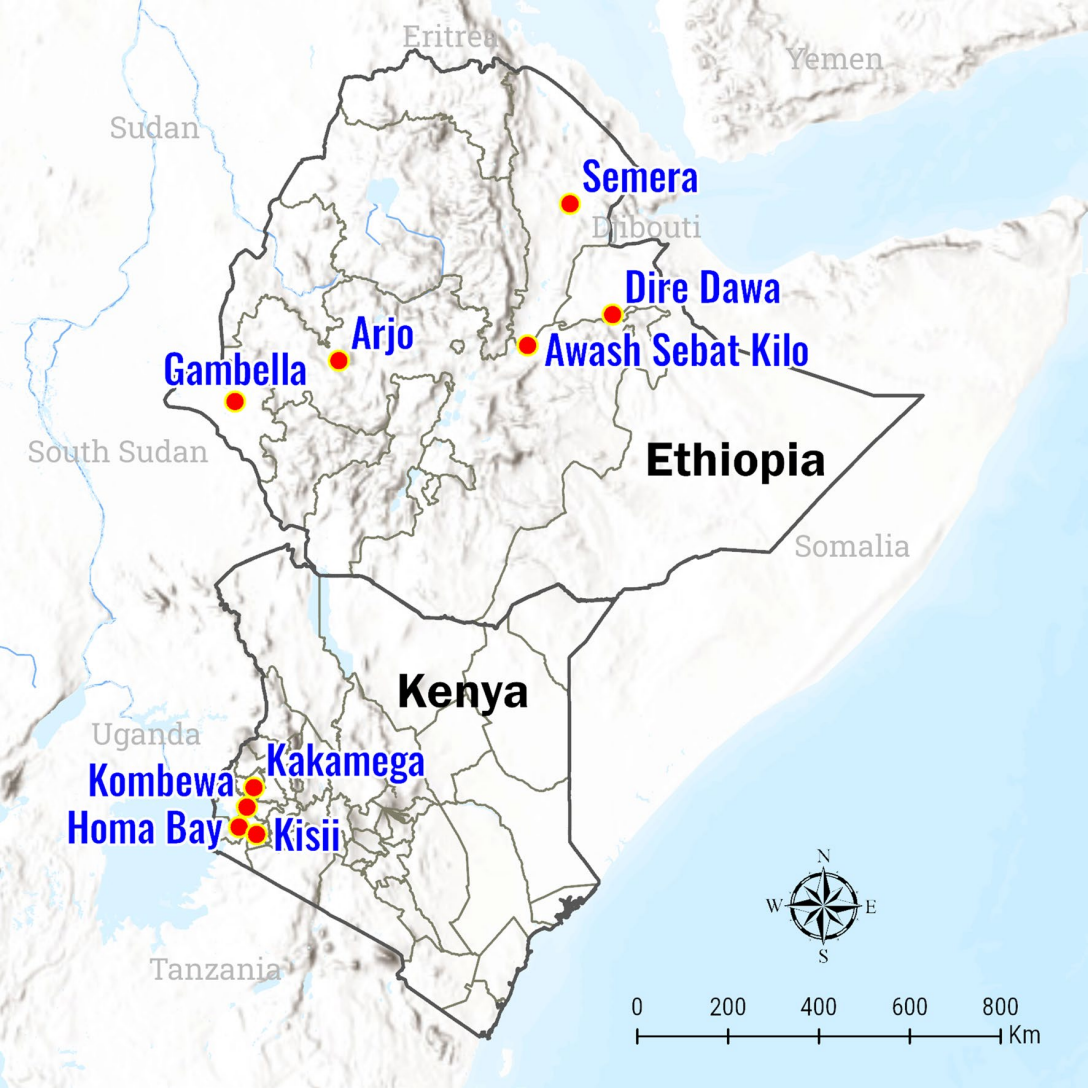
Map of study sites in Kenya (Homa Bay, Kakamega, Kisii, and Kombewa) and Ethiopia (Arjo, Awash, Dire Dawa, Gambella, and Semera)

Samples were collected from four study sites of varying transmission levels in western Kenya (Fig. 1). Kombewa (00°07′N, 34°30′E, 1,260 m above sea level [m a.s.l.]), in the lowland area of the Lake Victoria basin in Kisumu County, is a holoendemic site. Kakamega (00°10′N, 34°45′E, 1,500 m a.s.l.), in Kakamega County, represents the highland mesoendemic area. Kisii (00°35′S, 34°48′E, 1700 m a.s.l.) is a hypoendemic site in Kisii County. Homa Bay (0°31′S, 34°27′E, 1,190 m a.s.l), in southern Homa Bay County, represents the Lake mesoendemic transmission level. Parasite prevalence in Homa Bay County was historically greater than 20%, but indoor residual spraying campaigns conducted by the Kenyan Ministry of Health and the U.S. President’s Malaria Initiative since 2018 have reduced transmission significantly [22, 23]. At all sites, *P. falciparum* is the primary malaria parasite species. Western Kenya experiences a bimodal pattern of rainfall. January-March is the hot and dry season, April-June is the long rainy season, July-September is the cool and dry season, and October-December is the short rainy season.

Samples from five study sites across Ethiopia (Fig. 1), representing different transmission risk strata, as defined based on historical annual parasite incidence by Ethiopia’s National Malaria Control Strategy, were also analyzed [24]. Dire Dawa (9°36′N, 41°52′E, 1,280 m a.s.l.) in eastern Ethiopia and Arjo (8°42′N, 36°26′E, 1,350 m a.s.l.) in western Ethiopia are in the “very low risk” stratum. Semera (11°47′N, 41°0′E, 430 m a.s.l.) in northeastern Ethiopia and Hawassa (7°3′N, 38°28′E, 1700 m a.s.l.) in the central Ethiopian Rift Valley are in the “moderate risk” stratum. Gambella (7°53′N, 34°30′E, 500 m a.s.l.) in remote western Ethiopia is in the “high risk” stratum. *P. falciparum* and *P. vivax* are co-endemic in these sites. Ethiopia has three rainy seasons. The primary rainy season occurs from June-September, and a short rainy season occurs from February-May. Southern Ethiopia experiences a drier and colder third rainy season from October-December [25].

### Study design

The primary purpose of this study was to assess the overall prevalence of *k13* polymorphisms in study sites established under the National Institutes of Health’s sub-Saharan Africa International Centers of Excellence for Malaria Research (ICEMR) programme. Thus, dried blood spot samples (DBS) collected through ICEMR surveillance and research activities between 2018 and 2022 were investigated. Samples from four ICEMR study sites in Kenya (Homa Bay, Kakamega, Kisii, and Kombewa) and five ICEMR study sites in Ethiopia (Arjo, Awash, Dire Dawa, Gambella, Semera) were analysed (Table 1). Samples were identified through convenience sampling, therefore the number of samples per site and collection year was not balanced. Briefly, the sources of samples used in this study included passive case detection (PCD) at health centers, clinics, and hospitals; seasonal, cross-sectional mass blood surveys (MBS) in randomly selected households; schoolchildren dynamics studies, which seek to describe transmission dynamics among schoolchildren aged 5-18; and a three-year longitudinal cohort study with monthly follow-up. PCD captured cases of malaria that were confirmed via light microscopy or rapid diagnostic tests among community members who sought treatment at health facilities, while the remaining studies captured asymptomatic cases at households or schools within communities. For each study, DBS were collected via finger prick from all community members who consented to participating. DBS were stored with silica gel desiccant at 4 ºC until usage.

**Table 1:** ICEMR samples collected between 2018 and 2022 used for *Pfk13* polymorphism investigation

Kenya			Ethiopia		
Study site	Transmission intensity	Number of samples^ª^	Study site	Transmission Intensity	Number of samples
Homa Bay	Lake endemic (low)¶	463	Arjo	Low	91
Kombewa	Holoendemic	177	Gambella	High	133
Kakamega	Mesoendemic	123	Dire Dawa	Very low	50
Kisii	Hypoendemic	12	Semera	Moderate	22
			Awash	Moderate	23
Total		775	Total		319

All samples were collected as dried blood spots (DBS) via finger prick

^ª^Analyses were restricted to *P. falciparum* positive samples with Ct ≤ 32 by *var*ATS qPCR

^¶^Higher malaria transmission observed was observed in 2022 in Homa Bay compared to 2018-2021

### Parasite genotyping

DNA was extracted from DBS using the saponin/ Chelex method [26]. *Plasmodium falciparum* infections were identified by *var*ATS real-time quantitative PCR (qPCR) on a QuantStudio 3 Real-Time PCR System (Thermo Fisher Scientific, Carlsbad, CA) [27]. Isolates with cycle threshold (Ct) values ≤ 32 were selected to ensure sufficiently high parasitaemia for *k13* genotyping. In samples that met this Ct threshold, the *k13* propeller domain was amplified by nested PCR, using primers designed by Ariey et al*.* [3] and PerfeCTa qPCR ToughMix (QuantaBio, Beverly, MA). Cycling conditions and specifications of reaction mixtures are presented in Additional file 1: Table S1. Gel electrophoresis was performed at 120 V (2% agarose gel with ethidium bromide) on 5 µL of each nested PCR product, along with a 100 bp DNA ladder (New England Biolabs, Ipswich, MA), and gels were visualised under UV light to confirm amplification and correct amplicon size. For each successfully amplified sample, enzymatic cleanup on the remaining PCR product was performed using exonuclease I and shrimp alkaline phosphatase (Thermo Fisher Scientific). Purified PCR amplicons were screened for polymorphisms by Sanger sequencing in the forward direction (GENEWIZ, Inc., La Jolla, CA). For all samples with polymorphisms in the initial forward direction screening, fresh aliquots of DNA were used to prepare and clean PCR amplicons, following the steps described above. The purified PCR products were then sequenced in both directions to confirm polymorphisms.

### Data analysis

CodonCode Aligner 10.0.3 (CodonCode Corporation, Centerville, MA) was used to trim low-quality ends, assemble sequences, generate contigs, and align sequences. Sequences were exported and analysed using BioEdit 7.2 sequence alignment editor software [28]. Nucleotide and amino acid sequences were compared with the *P. falciparum* 3D7 chromosome 13 reference strain (GenBank Accession Number, CP017003.1). DNA Sequence Polymorphism (DnaSP) version 6 software [29] was used to first infer phased haplotypes for samples where heterozygous peaks were observed in sequencing chromatograms (e.g., presumed infection with two clones), and then to calculate population-level genetic variation indices, including the proportion of polymorphic loci, number of haplotypes, haplotype diversity, and nucleotide diversity. DnaSP was also used to perform Tajima’s and Fu and Li’s neutrality tests for all sites with mutant alleles (i.e., Homa Bay, Kakamega, and Kombewa in Kenya; Arjo, Dire Dawa, and Semera in Ethiopia). Haplotype sequences were deposited in NCBI GenBank under accession numbers OR571911-OR571926.

## Results

**Fig. 2:**
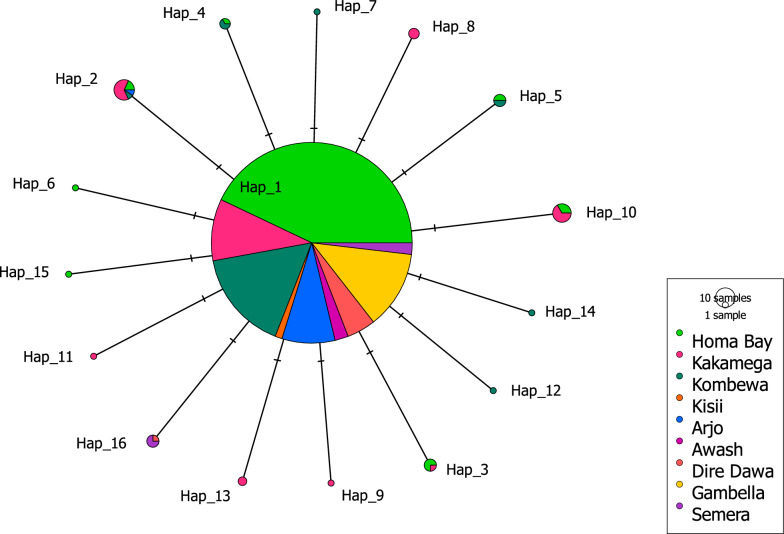
Haplotype network of *P. falciparum* isolates harbouring *k13* polymorphisms in study sites in Kenya and Ethiopia. The size of each circle corresponds to the haplotype prevalence. Each color represents the site where the haplotype was found; pie charts comprising multiple colors indicate the presence of the same haplotype in multiple study sites. Hap_1 represents the reference *k13* sequence (*Pf*3D7)

A total of 1,094 DBS between 2018 and 2022, comprising 775 DBS from Kenya and 319 DBS from Ethiopia, were successfully sequenced. Eight samples from Kenya showed multiclonal infections, as evidenced by double peaks in single nucleotide positions in the sequencing chromatograms. Thus, an additional eight sequences were obtained by phasing the isolates with heterozygote peaks, yielding 783 sequences from the Kenyan isolates. Sixteen unique haplotypes were identified from a total of 1,102 sequences analysed in this study. *k13* polymorphisms were detected in 42 samples from Kenya and five samples from Ethiopia (Table 2). Each mutant sample carried only one SNP in the *k13* propeller region, and only one haplotype (Hap_2, carrying the A582V mutation) was shared between the Kenyan and Ethiopian samples (Fig. 2). Among these samples, limited occurrences of two mutations that were added to the list of WHO-validated markers of artemisinin partial resistance in the 2022 World Malaria Report were observed [6]: A675V in Kenya and R622I in Ethiopia.

**Table 2:** Frequency and distribution of *Pfk13* propeller domain haplotypes in samples from Kenya and Ethiopia, 2018-2022

			Kenya, n (%)				Ethiopia, n (%)				
Haplotypes	Mutation	Type^†^	Homa Bay (N = 466)	Kakamega (N = 126)	Kombewa (N = 179)	Kisii (N = 12)	Arjo (N = 91)	Awash (N = 23)	Dire Dawa (N = 50)	Gambella (N = 133)	Semera (N = 22)
Hap_1	Wildtype	–	453 (97.21%)	105 (83.33%)	171 (95.53%)	12 (100%)	90 (98.90%)	23 (100.00%)	49 (98.00%)	133 (100.00%)	19 (82.61%)
Hap_2	A582V (1745C > T)	NS	2 (0.43)%	7 (5.56%)	1 (0.56%)	0	1 (1.10%)	0	0	0	0
Hap_3	V637V (1911 T > A)	S	3 (0.64%)	1 (0.79%)	0	0	0	0	0	0	0
Hap_4	G690G (2070C > G)	S	1 (0.21%)	0	2 (1.12%)	0	0	0	0	0	0
Hap_5	G533G (1599G > A)	S	2 (0.43)%	0	2 (1.12%)	0	0	0	0	0	0
Hap_6	H560H (1680 T > C)	S	1 (0.21%)	0	0	0	0	0	0	0	0
Hap_7	P655P (1965A > T)	S	0	0	1 (0.56%)	0	0	0	0	0	0
Hap_8	A675V (2024C > T)	NS	0	3 (2.38%)	0	0	0	0	0	0	0
Hap_9	P667S (1999C > T)	NS	0	1 (0.79%)	0	0	0	0	0	0	0
Hap_10	A578S (1732G > T)	NS	3 (0.64%)	6 (4.76%)	0	0	0	0	0	0	0
Hap_11	A569S (1705G > T)	NS	0	1 (0.79%)	0	0	0	0	0	0	0
Hap_12	I646L (1936A > T)	NS	0	0	1 (0.56%)	0	0	0	0	0	0
Hap_13	C469C (1407C > T)	S	0	2 (1.59%)	0	0	0	0	0	0	0
Hap_14	T535T (1605G > A)	S	0	0	1 (0.56%)	0	0	0	0	0	0
Hap_15	E643E (1929A > G)	S	1 (0.21%)	0	0	0	0	0	0	0	0
Hap_16	R622I (1865G > T)	NS	0	0	0	0	0	0	1 (2.00%)	0	3 (13.04%)

^†^*S* synonymous mutation, *NS* nonsynonymous mutation

The prevalence of nonsynonymous *k13* mutations in Kenya was 3.25%. The most common mutations overall among the Kenyan samples were A578S (9/783, 1.15%) and A582V (10/783, 1.28%). A578S appeared in Kakamega in 2019, 2021, and 2022 at increasing frequencies (Additional file 1: Table S3). Three samples collected in 2022 in Homa Bay also harboured the A578S mutation. A582V was observed at low frequencies in Homa Bay in 2018 (1.45%) and again in 2022 (0.64%). A675V occurred only in Kakamega in 2021, at a frequency of 25.00% (3/12). The nonsynonymous mutations A569S, I646L, and P667S were detected in only one sample each. Frequencies of synonymous *k13* polymorphisms in Kenya were low, ranging from 0.13-0.50%. Haplotype diversity was low in all four Kenyan study sites, with Hd ranging from 0 in Kisii, where no polymorphisms were detected, to 0.302 in Kakamega, where eight haplotypes were found. Nucleotide diversity was similarly low (π_i_ < 0.00015). Tajima’s D and Fu and Li’s F were negative for each population tested, congruous with the observation of an excess of rare alleles compared to the expected diversity under the assumptions of neutral selection and constant population size. However, both test statistics were only significant at a p-value < 0.05 for Homa Bay and Kombewa (Table 3).

**Table 3:** Molecular diversity of the *Pfk13* propeller gene in Kenya and Ethiopia, 2018-2022

							Tajima’s test of neutrality^†^		Fu and Li’s test of neutrality	
Country	Study site^§^	Sample size	Proportion of polymorphic sites	Number of haplotypes, h	Haplotype diversity, Hd ± SD	Nucleotide diversity, π_i_ ± SD (10^–3^)^¶^	Tajima's D	P-value	Fu and Li’s F test statistic	P-value
Kenya	Homa Bay	466	7/2181	8	0.055 ± 0.015	0.03 ± 0.01	−1.823	< 0.05	−2.345	< 0.05
	Kakamega	126	7/2181	8	0.302 ± 0.053	0.15 ± 0.03	−1.698	NS	−1.882	NS
	Kisii	12	0/2181	1	0	0	–	–	–	–
	Kombewa	179	6/2181	7	0.088 ± 0.029	0.04 ± 0.01	−1.885	< 0.05	−3.133	< 0.02
	Subtotal	738	14/2181	15	0.104 ± 0.015	0.05 ± n.d	−2.084	< 0.01	−3.771	< 0.02
Ethiopia	Arjo	91	1/2181	2	0.022 ± 0.021	0.01 ± 0.01	−1.038	NS	−1.999	NS
	Awash	23	0/2181	1	0	0	–	–	–	–
	Dire Dawa	50	1/2181	2	0.040 ± 0.038	0.02 ± 0.02	−1.103	NS	−1.884	NS
	Gambella	133	0/2181	1	0	0	–	–	–	–
	Semera	22	1/2181	2	0.247 ± 0.108	0.11 ± 0.05	−0.175	NS	0.479	NS
	Subtotal	319	2/2181	3	0.031 ± 0.014	0.01 ± 0.01	−1.150	NS	−1.499	NS
Total		1102	15/2181	16	0.083 ± 0.012	0.04 ± n.d	−2.088	< 0.01	−3.729	< 0.02

^¶^*n.d.* not determined by DnaSp v6 software

^§^Samples were aggregated by site (2018-2022) for molecular diversity calculations

^†^NS = not significant at P<0.05, “—” denotes no polymorphisms in population

The prevalence of nonsynonymous *k13* mutations in Ethiopia was 1.57%. One sample collected in Arjo in 2020 harboured the A582V mutation. Four occurrences of R622I were found among the 2022 samples: one sample from Dire Dawa and three samples from Semera. No synonymous *k13* mutations were detected in the 319 Ethiopian samples tested (Table 2). Site-level analyses revealed low haplotype diversity (Hd: 0-0.247) and nucleotide diversity (π_i_ < 0.00011). Negative Tajima’s D and Fu and Li’s F statistic values indicate an excess of singletons in Arjo and Dire Dawa (P > 0.05 for all neutrality tests). In Semera, Tajima’s D value was -0.17 (P > 0.05) and Fu and Li’s F value was 0.48 (P > 0.05), suggesting possible balancing selection of the R622I mutation in the study area.

## Discussion

In this study, the prevalence of synonymous and nonsynonymous mutations in 775 *P. falciparum* isolates collected from four sites in western Kenya and 319 *P. falciparum* isolates collected from five sites across Ethiopia between 2018 and 2022 was investigated. Low frequency of *k13* polymorphisms and few occurrences of shared alleles between geographically distinct populations were observed. These findings align with previous studies conducted in western Kenya [15, 16, 20, 30], Ethiopia [20, 21, 31], and across Africa [5, 20, 32, 33] following the implementation of ACT.

Seven unique nonsynonymous mutations were found in this study. A582V was the most common mutation among the Kenyan samples and is the only nonsynonymous mutation that was observed in both Kenya and Ethiopia. The mutation has been reported once among parasites collected across Kilifi County, Kenya between 1994 and 2018 [34]. A578S was the second most common mutation among the Kenyan samples in this study and is the most common *k13* polymorphism found across Africa [5]. An increase in the relative frequency of A578S mutants in Kakamega between 2019 and 2022 was observed, in line with a previous study conducted in Kombewa and Kakamega [30]. However, A578S is not associated with clinical or in vitro artemisinin partial resistance [5]. A675V, a recently validated marker of artemisinin partial resistance [6], appeared in three out of the 12 samples tested from Kakamega in 2021; the mutation was not detected at any study site in 2022. A569S, which appeared in only one 2019 sample, was reported in Kenya in 2013 in one sample from Mbita district, Homa Bay County [13] and one sample from Kisumu County [20]. I646L has been previously reported in Asia but has yet to be evaluated by the WHO or the Worldwide Antimalarial Resistance Network [35]. Another nonsynonymous mutation at the same codon, I646T, was detected in Sierra Leone in 2016, but the mutation similarly has not yet been evaluated for resistance [36]. In Rwanda, Straimer et al*.* reported parasite clearance time > 5 h. in a patient infected with *P. falciparum* parasites carrying the P667S mutation [37]. However, further investigation is required to confirm the potential impact of P667S on ring-stage survival in vitro.

R622I, which appeared in four PCD samples collected in 2022 in Ethiopia, is another recently validated marker of partial artemisinin resistance [38]. The mutation was first detected in samples from Northwest Ethiopia and increased in prevalence from 2.4% in 2014 to 9.5% in 2017-2018 [39]. Within Ethiopia, R622I has also been reported in samples from districts further north, along the border with Eritrea, and further south, along the border with South Sudan [40]. R622I has also been found in samples from Somalia, Sudan, Mozambique, Zambia, and Eritrea [38, 41]. In Eritrea, which shares a border with northern Ethiopia, the cumulative frequency of R622I in 729 samples collected between 2015 and 2020 was approximately 14% [6]. This study is the first to report cases of R622I in Semera (northeast Ethiopia), further underscoring the pressing need for monitoring clonal expansion of R622I across the country.

Samples were aggregated by study site for molecular diversity analyses in this study due to the limited number of *k13* polymorphisms found when samples were stratified by year and infection type. The imbalances in the number of samples analysed by site, year, and infection type reflect not only variations in transmission intensity by site but also differences in the ongoing activities among study sites. Most of the Kenyan samples investigated arose from asymptomatic infections (88.88%, 711/800), whereas most of the Ethiopian samples came from symptomatic infections detected at health facilities (92.16%, 294/319). In 2020, many health facilities were converted to treat patients afflicted with severe acute respiratory syndrome coronavirus 2 (SARS-CoV-2). Thus, the symptomatic samples used in this study were predominantly collected from health facilities in the years preceding the SARS-CoV-2 pandemic. On the other hand, passive surveillance remained largely uninterrupted in the study sites in Ethiopia, as evidenced by the symptomatic (i.e., PCD) samples available for use in this study, whereas community-based malaria surveys (i.e., asymptomatic samples) were pared down due to limited field team capacity and resources during the SARS-CoV-2 pandemic. No significant differences in the frequency or diversity of *k13* polymorphisms were observed between symptomatic and asymptomatic infections (Additional file 1: Tables S3, 4) when samples derived from both infection types were available for analysis, suggesting that the nature of infections did not clearly bias overall findings. However, the sample sizes available for comparison were small. Additionally, other studies have found that the extent to which symptomatic cases represent the asymptomatic parasite reservoir may vary with changes in transmission intensity [42, 43]. Therefore, spatiotemporally matched screening of both asymptomatic and symptomatic infections is necessary to evaluate potential sampling bias in molecular surveillance of artemisinin resistance.

The low number of mutations precluded statistical comparisons between years or study sites. Nevertheless, one key strength of this study was the optimization of available samples collected through various study sites and designs, which helped reduce confounding due to factors such as age and transmission intensity among the samples from Kenya. Several studies reporting low frequency of *k13* polymorphisms have focused exclusively on school-aged children [30, 44–46]. Despite constituting important drivers of malaria transmission, schoolchildren are frequently overlooked in malaria control programmes [47–50]. DBS were collected from asymptomatic school children aged 5-18 in Kakamega, Kisii, and Kombewa and from participants spanning the age spectrum in all studies conducted in Homa Bay (Additional file 1: Table S2). Interestingly, in nearly all studies conducted in Homa Bay, infections with high parasitemia (Ct ≤ 32) occurred most frequently in children 15 years old or younger. Furthermore, all 42 Kenyan samples carrying *k13* mutations (synonymous and nonsynonymous) occurred in children who were 4-15 years old. No associations between transmission intensity and nonsynonymous *k13* mutant allele frequency or diversity were observed. Taken together, these observations suggest that schoolchildren may be an appropriate sentinel group for artemisinin partial resistance surveillance in western Kenya, particularly when resources are limited. On the other hand, the age distribution among the Ethiopian samples tested for *k13* mutations more closely matched the age distribution of all enrolled participants within each study from which the samples were sourced. A higher frequency of R622I was observed in Semera compared to Dire Dawa (“moderate” vs. “very low” transmission sites, respectively). However, no *k13* mutations were found in Awash, which is similarly located in the “moderate risk” stratum. Surveillance strategies in Ethiopia might benefit from consideration of additional factors, aside from age and transmission intensity, in defining important sentinel groups and risk areas.

The variations in sample sizes also reflect differences in parasite density distributions by study sites and years. The number of samples tested for *k13* mutations in each study and year does not reflect the total number of samples collected but rather the number of samples with Ct ≤ 32. As this study was restricted to high density infections, rare mutant alleles present in low density infections may have been missed. Low density infections yield low concentrations of parasite DNA, rendering identification of minor alleles challenging to replicate. Additionally, low density infections often resolve without treatment and thus may have limited clinical relevance [51]. However, these infections may still contribute to malaria transmission and should be explored using more sensitive methods in future studies. Compared to next generation sequencing methods, Sanger sequencing, as used in this study, is less sensitive for detecting minor alleles in polyclonal infections [52]. Although the diversity of the parasite population is likely underestimated in this study, Sanger sequencing remains a highly accurate and rapid method for variant screening, and the use of more sensitive deep sequencing methods would sustain the overall trends reported here (no observations of clonal spread).

## Conclusion

An updated report on the low frequency of *k13* mutations in several study sites of western Kenya and across Ethiopia is presented in this study, in line with many other studies that have concluded that *k13* mutations remain rare in Africa. The *k13* validated marker A675V was restricted to three samples from asymptomatic schoolchildren in Kakamega, Kenya in 2019. In Ethiopia, four occurrences of the *k13* validated marker R622I were detected among symptomatic 2022 PCD cases from Dire Dawa and Semera, constituting one of the first reports of R622I from eastern Ethiopia. While ACT remains largely efficacious in these study sites in western Kenya and Ethiopia, enhanced molecular surveillance of *P. falciparum* resistance to artemisinin is necessary to monitor the spread of validated markers of resistance and the emergence of additional mutants of concern.

### Supplementary Information

**Additional file1: Table S1.** Nested *Pf*k13 PCR reaction mixtures and thermal cycling conditions. **Table S2.** Age distribution of samples tested for *Pfk13* polymorphisms. **Table S3.** Frequency of synonymous and nonsynonymous *Pfk13* polymorphisms in Kenya from 2018 to 2022, stratified by year and infection status. **Table S4.** Frequency of nonsynonymous *Pfk13* polymorphisms in Ethiopia from 2018 to 2022, stratified by year and infection status.

## Data Availability

The dataset generated in this study is available from the corresponding author upon reasonable request.

## Abbreviations

Pfk13: Kelch propeller domain on Plasmodium falciparum chromosome 13
DBS: Dried blood spot
SNP: Single nucleotide polymorphism
CQ: Chloroquine
SP: Sulfadoxine-pyrimethamine
AL: Artemether-lumefantrine
ACT: Artemisinin-based combination therapy
WHO: World Health Organization
Ct: Cycle threshold value
m a.s.l.: Metres above sea level
ICEMR: International Centers of Excellence for Malaria Research
PCD: Passive case detection
MBS: Mass blood surveys
varATS qPCR: Var gene acidic terminal sequence-based quantitative polymerase chain reaction
