# Supplementary material for: Molecular surveillance of Kelch 13 polymorphisms in Plasmodium falciparum isolates from Kenya and Ethiopia
Source: Malar J. 2024 Jan 29;23:36. doi: 10.1186/s12936-023-04812-y (PMC10823687; doi:10.1186/s12936-023-04812-y)
Supplement: Supplementary file 1 — Additional file1: Table S1. Nested Pfk13 PCR reaction mixtures and thermal cycling conditions. Table S2. Age distribution of samples tested for Pfk13 polymorphisms. Table S3. Frequency of synonymous and nonsynonymous Pfk13 polymorphisms in Kenya from 2018 to 2022, stratified by year and infection status. Table S4. Frequency of nonsynonymous Pfk13 polymorphisms in Ethiopia from 2018 to 2022, stratified by year and infection status. [file 12936_2023_4812_MOESM1_ESM.docx]

| **Additional Table 1.** Nested *Pf*k13 PCR reaction mixtures and thermal cycling conditions | | | | | | |
| --- | --- | --- | --- | --- | --- | --- |
| *Nest 1* | | | | | | |
| Reaction mixture | | Thermal cycling conditions | | | | |
| **Reagent** | **Volume (µl) per reaction** |  |  | **Temperature (ºC)** | **Time** | **Number of cycles** |
| PerfeCTa qPCR ToughMix (2X) | 6 | Step 1 | Initial Denaturation | 95 | 3min | 1 |
| Forward primer (10µM) | 0.5 | Step 2 | Denaturation | 94 | 30s | 35 |
| Reverse primer (10µM) | 0.5 |  | Annealing | 50 | 30s |  |
| Template DNA | 2 |  | Extension | 68 | 1.5min |  |
| Nuclease-free water | 3 | Step 3 | Final extension | 72 | 6 | 1 |
| Total | 12 | Step 4 | Hold | 10 | ∞ |  |
|  |  |  |  |  |  |  |
| *Nest 2* | | | | | | |
| Reaction mixture | | Thermal cycling conditions | | | | |
| **Reagent** | **Volume (µl) per reaction** |  |  | **Temperature (ºC)** | **Time** | **Number of cycles** |
| PerfeCTa qPCR ToughMix (2X) | 8.5 | Step 1 | Initial Denaturation | 95 | 3min | 1 |
| Forward primer (10µM) | 0.5 | Step 2 | Denaturation | 94 | 30s | 30 |
| Reverse primer (10µM) | 0.5 |  | Annealing | 55 | 30s |  |
| Nest 1 PCR product | 2 |  | Extension | 72 | 1min |  |
| Nuclease-free water | 5.5 | Step 3 | Final extension | 72 | 6min | 1 |
| Total | 17 | Step 4 | Hold | 10 | ∞ |  |

| **Additional Table 2.** Age distribution of samples tested for *Pfk13* polymorphisms | | | | | |
| --- | --- | --- | --- | --- | --- |
| *Study Site* | *Study Type* | *Study Period* | *Mean age of participants enrolled (IQR)^†^* | *Number of samples analyzed for k13 mutations^•^* | *Mean age of participants in samples analyzed (IQR)* |
| *Kenya* | | | | | |
| Homa Bay | Cohort | 2019–2022 | 19.57 (7­–28) | 266 | 10.52 (6–12) |
|  | MBS | 2018–2021 | 24.48 (7–37) | 108 | 15.04 (8–15) |
|  | PCD | 2018, 2019 | 18.23 (4–25) | 89 | 12.93 (5.13–16) |
| Kakamega | Dynamics | 2021–2022 | 9.63 (8–11) | 123 | 11.16 (10–13) |
| Kisii | Dynamics | 2021 | 8.26 (7–10) | 12 | 11.58 (10–14) |
| Kombewa | Dynamics | 2018–2019, 2021–2022 | 10.98 (10–13) | 177 | 11.37 (10–13) |
| *Ethiopia* | | | | | |
| Arjo | PCD | 2018–2020 | 24.57 (16–32) | 91 | 24.66 (19.5–32.5) |
| Gambella | MBS | 2018–2020 | 21.98 (9–30) | 25 | 16.64 (7–23) |
|  | PCD | 2018–2021 | 20.49 (9–29) | 108 | 17.83 (9.25–23.75) |
| Dire Dawa | PCD | 2022 | 21.35 (8–30) | 50 | 26.78 (16–31.75) |
| Semera | PCD | 2022 | 18.96 (6.5–27) | 22 | 19.73 (7.5–26.5) |
| Awash | PCD | 2022 | 27.73 (18–35) | 23 | 29.52 (18.5–35.5) |
| ^†^IQR = interquartile range (25^th^–75^th^ percentile)  ^*^Samples with *P. falciparum* qPCR Ct≤32 | | | | | |

| **Additional Table 3.** Frequency of synonymous and nonsynonymous *Pfk13* polymorphisms in Kenya from 2018–2022, stratified by year and infection status | | | | | | | | | | | | | | | | | | |
| --- | --- | --- | --- | --- | --- | --- | --- | --- | --- | --- | --- | --- | --- | --- | --- | --- | --- | --- |
|  | 2018 | | |  | 2019 | | | | |  | 2021 | | | |  | 2022 | | |
|  | Homa Bay | | Kombewa |  | Homa Bay | | Kakamega | Kombewa | Kisii |  | Homa Bay | Kakamega | Kombewa | Kisii |  | Homa Bay | Kakamega | Kombewa |
| Synonymous mutations | S^†^  (n=40) | AS  (n=69) | AS  (n=46) |  | S  (n=49) | AS  (n=43) | AS  (n=106) | AS  (n=48) | AS  (n=6) |  | AS  (n=59) | AS  (n=12) | AS  (n=64) | AS  (n=6) |  | AS  (n=156) | AS  (n=5) | AS  (n=21) |
| V637V  (1911T>A) | — | 1  (1.45%) | — |  | — | — | — | — | — |  | — | 1  (8.33%) | — | — |  | 2  (1.28%) | — | — |
| G533G  (1599T>A) | — | — | 1  (2.17%) |  | — | 2  (4.65%) | — | 1  (2.08%) | — |  | — | — | — | — |  | — | — | — |
| H560H  (1680T>C) | — | — | — |  | — | 1  (2.33%) | — | — | — |  | — | — | — | — |  | — | — | — |
| C469C  (1407C>T) | — | — | — |  | — | — | — | — | — |  | — | 1  (8.33%) | — | — |  | — | 1  (20.00%) | — |
| T535T  (1605G>A) | — | — | — |  | — | — | — | — | — |  | — | — | 1  (1.56%) | — |  | — | — | — |
| P655P  (1965A>T) | — | — | 1  (2.17%) |  | — | — | — | — | — |  | — | — | — | — |  | — | — | — |
| G690G  (2070C>G) | 1  (2.50%) | — | 1  (2.17%) |  | — | — | — | 1  (2.08%) | — |  | — | — | — | — |  | — | — | — |
| E643E  (1929A>G) | — | — | — |  | — | — | — | — | — |  | — | — | — | — |  | 1  (0.64%) | — | — |
| Nonsynonymous mutations |  |  |  |  |  |  |  |  |  |  |  |  |  |  |  |  |  |  |
| A569S  (1705G>T) | — | — | — |  | — | — | 1  (0.94%) | — | — |  | — | — | — | — |  | — | — | — |
| A578S  (1732G>T) | — | — | — |  | — | — | 3  (2.83%) | — | — |  | — | 2  (16.67%) | — | — |  | 3  (1.92%) | 1  (20.00%) | — |
| A582V  (1745C>T) | — | 1  (1.45%) | — |  | — | — | 7  (6.60%) | 1  (2.08%) | — |  | — | — | — | — |  | 1  (0.64%) | — | — |
| I646L  (1936A>T) | — | — | — |  | — | — | — | — | — |  | — | — | — | — |  | — | — | 1  (4.76%) |
| P667S  (1999C>T) | — | — | — |  | — | — | 1  (0.94%) | — | — |  | — | — | — | — |  | — | — | — |
| A675V  (2024>T) | — | — | — |  | — | — | — | — | — |  | — | 3  (25.00%) | — | — |  | — | — | — |
| ^†^S=symptomatic infections, AS=asymptomatic infections | | | | | | | | | | | | | | | | | | |

| **Additional Table 4.** Frequency of nonsynonymous *Pfk13* polymorphisms in Ethiopia from 2018–2022, stratified by year and infection status | | | | | | | | | | | | | | | | | |
| --- | --- | --- | --- | --- | --- | --- | --- | --- | --- | --- | --- | --- | --- | --- | --- | --- | --- |
|  | 2018 | | |  | 2019 | | |  | 2020 | | |  | 2021 |  | 2022 | | |
|  | Arjo | Gambella | |  | Arjo | Gambella | |  | Arjo | Gambella | |  | Gambella |  | Awash | Dire Dawa | Semera |
| Nonsynonymous mutations | S^†^  (n=32) | S  (n=29) | AS  (n=1) |  | S  (n=32) | S  (n=17) | AS  (n=10) |  | S  (n=27) | S  (n=35) | AS  (n=14) |  | S  (n=27) |  | S  (n=23) | S  (n=50) | S  (n=22) |
| A582V  (1745C>T) | — | — | — |  | — | — | — |  | 1  (3.70%) | — | — |  | — |  | — | — | — |
| R622I  (1865G>T) | — | — | — |  | — | — | — |  | — | — | — |  | — |  | — | 1  (2.00%) | 3  (13.64%) |
| ^†^S=symptomatic infections, AS=asymptomatic infections | | | | | | | | | | | | | | | | | |
